# A qualitative and quantitative cross‐sectional study on the past, present, and future of vaginal delivery: Turkey

**DOI:** 10.1002/ijgo.15849

**Published:** 2024-08-16

**Authors:** Fatma Keskin

**Affiliations:** ^1^ Department of History of Medicine and Ethics, Cerrahpasa Faculty of Medicine Istanbul University Istanbul Turkey

**Keywords:** breech presentation, future obstetrics, macrosomic fetus, residency training, twin pregnancy, vaginal delivery

## Abstract

**Objective:**

The current study focused on predicting future trends in obstetrics by examining obstetricians' approaches to specific challenging vaginal delivery that require special experience, such as breech presentation, macrosomic fetus, twin pregnancy, and vacuum use, compared with their residency training experience.

**Methods:**

The cross‐sectional study was conducted in two phases. The first phase was qualitative and the second phase was quantitative. The “interview” and “survey” techniques served as data collection tools. In total, 20 obstetricians participated in the interviews, and 400 obstetricians took part in the survey. Data from the interviews were analyzed using the Maxqda 2020 qualitative data analysis program, and survey data were analyzed using SPSS version 25.0.

**Results:**

Over the past 2 decades, there has been a gradual shift from vaginal deliveries to cesarean deliveries in cases involving breech presentation, macrosomic fetus, twin pregnancy, and vacuum use. While medicolegal concerns are undeniable, the prevalent belief among obstetricians that cesarean delivery is safer than vaginal delivery significantly influences this trend. Comparatively, young obstetricians often complete their residency training without acquiring sufficient knowledge and skills in vaginal delivery.

**Conclusion:**

Young obstetricians currently lack adequate experience in managing vaginal deliveries for breech presentation, macrosomic fetus, twin pregnancy, and vacuum use. This experience is at risk of disappearing entirely within the next decade as senior obstetricians retire. Policymakers should take this into consideration when shaping future healthcare policies.

## Synopsis

The gradual disappearance of experience in vaginal delivery from residency training highlights a critical need to reintroduce this aspect over the next decade.

## 1 INTRODUCTION

Turkey has one of the highest cesarean delivery rates globally, reaching 58.4%.^1^ This prevalence is largely influenced by obstetricians' inclination toward cesarean delivery, driven by medicolegal concerns.^2^ Furthermore, the development of patient rights has granted mothers a significant role in the decision‐making process.^3^ Despite the implementation of various national health policies aimed at reducing high cesarean delivery rates, the desired success remains elusive. This circumstance raises questions about the future trajectory of obstetrics. The fact that cesarean delivery rates exceed vaginal delivery rates highlights the danger of the vaginal delivery experience disappearing from obstetric practice.

Therefore, we herein analyzed obstetricians' approaches to vaginal deliveries necessitating specialized expertise, such as breech presentation, macrosomic fetus, twin pregnancies, and vacuum use. A comparative assessment with their residency training was conducted, and predictions were formulated regarding the future of obstetrics. The anticipated outcome of this research is to provide insights that can contribute to initiatives aimed at reducing cesarean delivery rates and enhancing maternal and infant health in the future.

## 2 MATERIALS AND METHODS

This qualitative and quantitative cross‐sectional study was conducted between October 2019 and June 2021 in accordance with the 1975 Declaration of Helsinki, following the approval of the ethics committee of Sakarya University Non‐Invasive Clinical Research (October 2, 2019; approval number: 71522473/050.01.04/125).

Written informed consent was obtained from all participants. STROBE (Strengthening the Reporting of Observational Studies in Epidemiology) guidelines were followed in the study.

The study was designed in two phases: The first phase was qualitative, and the second phase was quantitative. Interview and survey techniques were employed as data collection methods. Obstetricians who completed their residency training and were actively working were included in the study. The first phase of the study was performed between October 15, 2019, and January 12, 2020, at Sakarya University Faculty of Medicine and affiliated hospitals in Sakarya, located in the Marmara Region. In this phase, face‐to‐face, open‐ended, and semistructured interviews were conducted with 20 obstetricians in their natural working environment. All participants signed an informed consent form that was read to them before the interview. The goal was to comprehend the perceptions of obstetricians regarding both vaginal and cesarean deliveries. The current approach of obstetricians to breech presentation, macrosomic fetuses, twin pregnancies, and vacuum use was compared with their approach during residency training, and predictions were made concerning the future of obstetrics. The collected data were analyzed using the MAXQDA 2020 for Windows (Verbi, Comp) qualitative data analysis program.

In the second phase, an anonymized survey was developed by organizing and simplifying the data obtained from the interview results. The survey aimed to validate the findings of the interviews and derive general results. It was conducted in the Marmara region, which houses one‐third of the country's obstetricians and best reflects the demographic, social, and cultural structure of Turkey because of its cosmopolitan nature.

The sample size was determined as 322, using a 95% confidence interval and 5% error tolerance. The study was completed with the participation of 400 obstetricians between August 1, 2020, and June 30, 2021. The cluster sampling method, one of the probability sampling methods, was used as the sampling method in the study.

The survey initially started face‐to‐face but was later continued online and in person following the decision of the ethics committee because of the COVID‐19 epidemic. Before starting to answer, participants read an information section added to the survey and checked a box provided for their consent.

Data obtained from the survey were analyzed using SPSS Statistics for Windows, version 25.0 (IBM). Descriptive statistical methods (frequency and percentage) were employed to assess the prevalence of obstetricians' opinions regarding vaginal and cesarean delivery. Percentage and frequency were also used to evaluate the prevalence of obstetricians' approaches to breech presentation, macrosomic fetuses, twin pregnancies, and vacuum use in their current practice and residency training. The relationship between obstetricians' approaches to breech presentation, macrosomic fetuses, twin pregnancies, and vacuum use in their residency training and their length of expertise was examined using *χ* ^2^ test. A *P* value < 0.05 was considered statistically significant.

## 3 RESULTS

In the interview phase of the study, 20 obstetricians participated, with an average age of 41.9 years and an average expertise of 10.7 years. The survey involved 400 obstetricians, averaging 44.5 years of age and 14.3 years of expertise. The opinions of obstetricians regarding vaginal and cesarean delivery were explored through interview and survey questions, yielding the following results.

During the interviews, obstetricians generally characterized vaginal delivery as a prolonged, dynamic, and unpredictable process. In contrast, they described cesarean delivery as a more controllable and relatively safe procedure. All obstetricians reported experiencing varying levels of stress, fear, anxiety, tension, loneliness, and feelings of inadequacy during the vaginal delivery follow‐up process. Unforeseen complications of vaginal delivery and concerns about litigation were identified as the main stressors, a sentiment supported by the survey results. Of the surveyed obstetricians, 348 (87.1%) defined the stress level during vaginal delivery follow‐up as “very high” and “high.” In addition, 250 (62.5%) obstetricians stated that the fear of litigation significantly influenced their cesarean delivery decisions at “very high” and “high” levels. Only 23 (5.8%) stated that the concern about being sued did not affect their cesarean delivery decisions. Moreover, 15 of the obstetricians interviewed stated that they felt safer during cesarean delivery, one stated that he felt safer during vaginal delivery, two stated that they felt safe in both types of delivery, and two did not feel safe in both types of delivery. Obstetricians felt safer during the cesarean delivery because they were in control, and there were fewer unexpected complications. In the survey, 14 (3.5%) obstetricians felt safer in vaginal delivery, while 180 (45%) felt safer in cesarean delivery. A notable 96 (24.0%) felt safe in both types of delivery, and 110 (27.5%) did not feel safe in either.

Eight of the obstetricians interviewed stated that they considered cesarean delivery to be safer for both the mother and baby than vaginal delivery, whereas 10 obstetricians expressed the opposite view. Two obstetricians noted that they perceived cesarean delivery as safer for the baby but not for the mother. In terms of the survey results, 182 (45.5%) obstetricians deemed cesarean delivery safer for both the mother and the baby compared with vaginal delivery. A total of 121 (30.3%) indicated that they found it safer for the baby but not for the mother. The remaining 81 (20.3%) did not consider cesarean delivery to be safer for both parties.

Obstetricians' approaches to breech presentation, macrosomic fetus, twin pregnancy, and vacuum use were examined compared with their residency training experience, revealing the following results.

**FIGURE 1:**
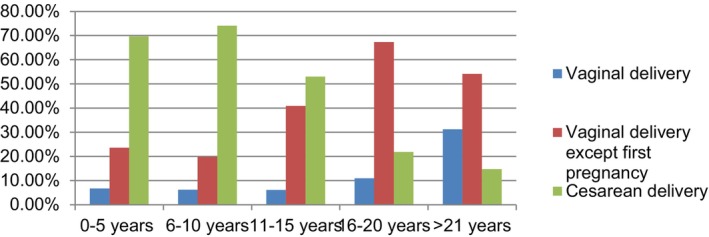
The relationship between the approach to breech presentation in obstetricians' residency training and the length of expertise (*χ* ^2^ = 119.162; *P* < 0.001 and <0.05).

While the obstetricians interviewed acknowledged that breech presentation was not necessarily an indication for cesarean delivery, they preferred cesarean delivery to avoid potential intervention and complications. Those with 14 years or more of experience indicated that vaginal delivery was preferred for breech presentation in their residency training, but, over time, they increasingly opted for cesarean delivery because of medicolegal concerns. Obstetricians with less than 5 years of experience reported that their residency training emphasized cesarean delivery for breech presentation, and they continued this practice in their professional lives because of insufficient experience in managing vaginal breech delivery. The survey results detailing obstetricians' preferences for delivery mode in breech presentation are summarized in Table 1, comparing them with the general trend in residency training. The results depicted in Figure 1 illustrate a shift from vaginal delivery to cesarean delivery in breech presentation during residency training over the past 20 years.

**TABLE 1:** Obstetricians' approaches to breech presentation in their residency training and current practice.

Approaches to breech presentation	Residency training		Current practice	
Vaginal delivery	*n* = 55	13.8%	*n* = 12	3%
Vaginal delivery except first pregnancy	*n* = 160	40%	*n* = 81	20.3%
Cesarean delivery	*n* = 185	46.3%	*n* = 307	76.8%
Total	*N* = 400	100%	*N* = 400	100%

**FIGURE 2:**
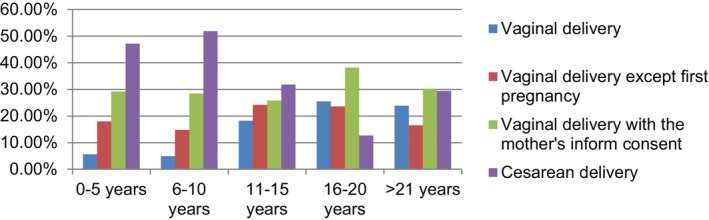
The relationship between the approach to a 4000g fetus in obstetricians' residency training and the length of expertise (*χ* ^2^ = 43.625; *P* < 0.001 and <0.05).

Another noteworthy outcome from the study was obstetricians' preference for cesarean delivery when the intrauterine fetal weight exceeded 4000 g. Despite acknowledging the recommended weight thresholds of 4500 g for pregnant women with diabetes and 5000 g for those without diabetes, as per the guidelines, the interviewed obstetricians stated a preference for cesarean delivery for fetuses weighing 4000 g and above. This preference stemmed from concerns about increased intervention and complications. Obstetricians with 10 years or more of professional experience indicated that, during their residency training, vaginal delivery was favored for macrosomic fetuses if the mother's examination findings were appropriate. However, over time, they increasingly opted for cesarean delivery because of medicolegal concerns. On the other hand, obstetricians with less than 10 years of professional experience mentioned that during their residency training, babies weighing 4000 g and above were delivered by cesarean section and that they continued this practice in their professional lives. Table 2 provides additional support for these findings, as reflected in the survey results. Figure 2 illustrates the evolution of the approach to delivering 4000g fetuses in residency training over the past 20 years.

**TABLE 2:** Obstetricians' approaches to a 4000g fetus in their residency training and current practice.

Approaches to a 4000g fetus	Residency training		Current practice	
Vaginal delivery	*n* = 61	15.3%	*n* = 24	6.0%
Vaginal delivery except first pregnancy	*n* = 75	18.8%	*n* = 55	13.8%
Vaginal delivery with the mother's informed consent	*n* = 120	30.0%	*n* = 139	34.8%
Cesarean delivery	*n* = 144	36.0%	*n* = 182	45.5%
Total	*N* = 400	100%	*N* = 400	100%

**FIGURE 3:**
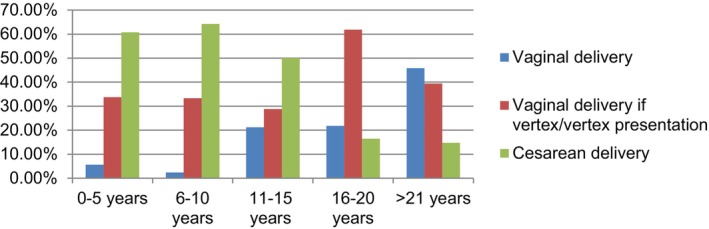
The relationship between the approach to twin pregnancies in obstetricians' residency training and the length of expertise (*χ* ^2^ = 113.518; *P* < 0.001 and <0.05).

A parallel pattern was observed in obstetricians' approaches to delivery in twin pregnancies. Among the physicians interviewed, those with 10 years or more of professional experience mentioned that, in their residency training, twin pregnancies were delivered vaginally, especially in vertex/vertex presentation. However, with time, they shifted toward cesarean delivery, primarily because of medicolegal concerns. Obstetricians with less than 5 years of professional experience indicated that, during their residency training, twin pregnancies were predominantly delivered by cesarean section, and they continued this practice in their professional lives. Table 3 offers a comprehensive overview that summarizes the survey results in comparison with the general trend observed in residency training. Figure 3 illustrates a shift in the approach to twin pregnancies from vaginal delivery to cesarean delivery in residency training over the past 20 years.

**TABLE 3:** Obstetricians' preferences for delivery mode in twin pregnancies in their residency training and current practice.

Approaches to twin pregnancies	Residency training		Current practice	
Vaginal delivery	*n* = 83	20.8%	*n* = 29	7.3%
Vaginal delivery if vertex/vertex presentation	*n* = 153	38.3%	*n* = 114	28.5%
Cesarean delivery	*n* = 164	41.0%	*n* = 257	64.3%
Total	*N* = 400	100%	*N* = 400	100%

**FIGURE 4:**
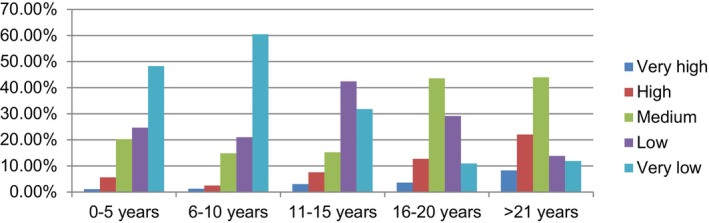
The relationship between vacuum usage rates in obstetricians' residency training and the length of expertise (*χ* ^2^ = 43.625; *P* < 0.001 and <0.05).

Concerning vacuum use, senior obstetricians in the interviews mentioned that they employed vacuum for appropriate indications during their residency training. However, they currently refrain from using it in their practice because of associated complications, opting instead for cesarean delivery, particularly in the early phases before considering vacuum use. This decision is influenced by medicolegal concerns. Conversely, young obstetricians stated that vacuum was not a preferred method during their residency training. They expressed reluctance to use it in their own practice and preferred cesarean delivery when faced with situations requiring vacuum assistance. Table 4 provides a concise summary of obstetricians' opinions on vacuum use, drawing a comparison with their experiences during residency training, as indicated by survey results. Figure 4 provides a summary of vacuum usage rates during obstetricians' residency training over the past 20 years.

**TABLE 4:** Vacuum usage rates of obstetricians in their residency training and current practice.

Vacuum usage rates	Residency training		Current practice	
Very high	*n* = 15	3.8%	*n* = 1	0.3%
High	*n* = 43	10.8%	*n* = 8	2.0%
Medium	*n* = 112	28.0%	*n* = 70	17.5%
Low	*n* = 98	24.5%	*n* = 115	28.8%
Very low	*n* = 132	33.0%	*n* = 206	51.5%
Total	*N* = 400	100%	*N* = 400	100%

In addition to these insights, 17 of the interviewed obstetricians and 283 (70.8%) of the survey respondents expressed the view that the 15% cesarean delivery rate recommended by the World Health Organization (WHO) in 1985 is unrealistic under present‐day conditions. The survey results further outline obstetricians' opinions on the ideal cesarean delivery rate in contemporary circumstances, as presented in Table 5.

**TABLE 5:** Opinions of obstetricians about the ideal cesarean delivery rate under today's conditions.

Ideal cesarean delivery rates	Number	Percentage
15%–20%	35	8.8
20%–25%	45	11.3
25%–30%	79	19.8
30%–35%	95	23.8
35%–40%	146	36.5
Total	400	100

## 4 DISCUSSION AND CONCLUSIONS

The field of obstetrics is currently undergoing a visible transformation process, a phenomenon not new but significantly accelerated in the past 2 decades. Historically, before the 19th century, cesarean sections were performed as a last resort for a deceased or dying mother, aiming to save the baby in cases where vaginal delivery was impossible. Such situations included a severely narrow pelvis, tumors obstructing the delivery path, or fetal anomalies. Advances such as anesthesia and antiseptics in the 19th century, along with the introduction of blood groups and safe blood transfusions in the early 20th century, contributed to the increased safety of cesarean delivery, and it has become a safe option in cases of heavy bleeding such as placenta previa and placental abruption.^4^ By the 1960s, antibiotics further reduced the difference in mortality and morbidity between cesarean and vaginal deliveries.^5^ Finally, in 2000, the Term Breech Trial published its findings, asserting that cesarean delivery was safer than vaginal delivery in cases of breech presentation.^6^ Despite conflicting findings, the Term Breech Trial significantly influenced the preference for cesarean deliveries in breech presentations.^7^ Although it received intense criticism at the time, this study's results indicate that obstetricians are increasingly adopting this trend.

Currently, vaginal delivery is perceived as an uncertain process with unpredictable outcomes, contrasting with the perception of cesarean delivery as a relatively more controlled and safe operation. Obstetricians' opinions on ideal cesarean delivery rates exceed the recommendations of the WHO and closely align with the rates targeted for reduction.^8^, ^9^ The study findings are consistent with Molina et al.'s findings in 2015 that the optimal cesarean rate recommended by the WHO is a very high target. However, the fact that it is much higher than the 19% optimal cesarean section rate recommended by them is concerning for the future of obstetrics.^10^

This shift challenges the traditional acceptance that vaginal delivery is safer for both the mother and baby than cesarean delivery, with an increasing consensus among obstetricians that cesarean delivery is the safer option.

Undoubtedly, obstetricians' preference for cesarean delivery because of medicolegal concerns has played a pivotal role in expediting this transformation.^2^, ^11^ The results of this study support the results of previous studies on this subject, but also indicate that this trend is especially decisive for senior obstetricians. This trend has also contributed to permanent changes in residency training by increasing cesarean section rates. The consequence of the rising cesarean delivery rates and declining vaginal delivery rates is the diminishing experience of vaginal delivery during residency training. Senior obstetricians tend to avoid vaginal delivery primarily because of medicolegal concerns. Conversely, young obstetricians consider breech presentation, a fetus weighing 4000 g, and twin pregnancies as indications for cesarean delivery. Vacuum use has also been overshadowed by cesarean delivery in contemporary obstetric practice.^12^, ^13^

This trend justifies concerns that young obstetricians are completing their residency training without acquiring sufficient knowledge and skills regarding the management of complications during vaginal delivery.

In vaginal delivery management guidelines, it is particularly emphasized that the obstetrician performing vaginal delivery in breech presentation, twin pregnancies, and macrosomic fetuses must be experienced; otherwise, cesarean delivery is recommended. Obtaining the mother's informed consent is also highlighted.^14^, ^15^, ^16^ The lack of sufficient experience in vaginal delivery makes cesarean delivery a safer option for both the obstetrician and the patient, at least in the short term.

In this scenario, it is foreseeable that in the next 10 years, as senior obstetricians gradually withdraw from working life, all vaginal deliveries requiring expertise will be replaced by cesarean delivery. This shift may result in a further increase in cesarean delivery rates and related complications in the long run.^17^, ^18^ Therefore, reintroducing vaginal deliveries requiring special expertise into residency training holds a crucial role in reducing cesarean delivery rates and planning studies on the health of both mother and baby in the future.^19^, ^20^, ^21^ The next decade presents significant opportunities to reintroduce the experience in vaginal delivery, which, although still existing in the field of obstetrics, remains underutilized for various reasons, into residency training.

In conclusion, the future of obstetrics lies in striking a balance between advancing medical interventions and preserving the invaluable expertise of vaginal deliveries. Efforts to reintroduce specialized experiences into residency training can shape a more holistic and patient‐centered approach to maternal health care, fostering a positive impact on the well‐being of both mothers and infants.

## AUTHOR CONTRIBUTIONS

Fatma Keskin is the sole author.

## FUNDING INFORMATION

No funding has been received for the conduct or publish of the study.

## CONFLICT OF INTEREST STATEMENT

The author has no conflicts of interest.

## Data Availability

Data sharing is not applicable to this article as no new data were created or analyzed in this study.
